# Monthly Memoranda

**Published:** 1854-06

**Authors:** 


MONTHLY MEMORANDA.

*Maternal Influence.*—A remarkable case of this nature recently occurred in New Hampshire, and is reported to the Amer. Lancet, by Rev. Mr. Hartwell, and if true, as the position of the reporter would seem to indicate, is peculiarly interesting in a physiological point of view. It seems that a Mr. Kimball was the proprietor of a domestic animal commonly known as a cat, who was usually very fond of society. Said social cat, however, when attentions were shown her by a little girl with a club foot, seemed very much frightened particularly on account of the distorted limb, for Mrs. Cat resisted all the kindness offered by her young visitor, and on seeing the foot, would make the most desperate efforts to escape and hide. But the *cat*astrophe is, that about six weeks after the girl's visit, Madame Feline was brought to bed, when she was endowed with four promising kittens, each one of whom had *one* foot turned in, precisely like the girls', and the *same* foot in every puss. The case is gravely reported and seems to be true. It appears to prove clearly that maternal influences do effect future generations. Another wonder in this case seems to be that her catship should have been of so observing a turn of mind, as to be so deeply impressed with the unfortunate condition of her young admirer.

*Replacing dislocated teeth.*—Dr. Stone, of Canada, reports that a blacksmith, by an unlucky blow, had two teeth knocked quite out of his mouth upon the floor. After the lapse of half an hour, he found Dr. S. and insisted upon having the teeth replaced. This was done, bandages were applied and the union became firm and permanent.

*How soon will a Drowned Body Float?*—A question of some *medico-legal* interest was lately tried at New York. Mr. C. S. had his life insured for $10,000, and policy was issued May 15, 1850, premium payable quarterly in advance. On August 23, 1850, C. S. paid the premium for the quarter ending Nov. 15. Now, the plaintiffs, the assignees of the policy, alledge that on September 4th, C. S. was drowned, while fishing with two other persons, in New York bay.—The defendants, who issued the policy, say that the three persons had entered into a conspiracy to defraud the Insurance Company, by insuring the life of C. S. for a large amount, then secreting and disposing of him. To obtain a recovery the plaintiffs must satisfy the jury of the death of C. S. This they do. first by the testimony of Ottman, one of his companions, who swore to all the facts. Second, by showing that a body, found floating near Jersey City, on September 7, was the body of C. S., which was examined by the Coroner when found, and then buried. The identity is proved mainly by particulars respecting the dress. The defence is, by showing from numerous witnesses, coroners and physicians, that bodies do not rise and float under from six to ten days; and as C. S. was alledged to be drowned on the 4th, and the body floated on the 7th, the body could not be that of C. S. Four witnesses were called by the defense, who agreed that the time depends on the size of the body, season of year, and temperature of the water; and all agreed that they do not usually rise in less than seven or ten days, even in summer. Four others are called by plaintiffs, experienced coroners, who had known cases of rising in two or three days, and even the next day, and who represented floating in three days as quite common. The verdict of the jury was in favor of plaintiffs.

